# Apolipoprotein A-I modulates HDL particle size in the absence of apolipoprotein A-II

**DOI:** 10.1016/j.jlr.2021.100099

**Published:** 2021-07-27

**Authors:** John T. Melchior, Scott E. Street, Tomas Vaisar, Rachel Hart, Jay Jerome, Zsuzsanna Kuklenyik, Noemie Clouet-Foraison, Carissa Thornock, Shimpi Bedi, Amy S. Shah, Jere P. Segrest, Jay W. Heinecke, W. Sean Davidson

**Affiliations:** 1 Center for Lipid and Arteriosclerosis Science, Department of Pathology and Laboratory Medicine, University of Cincinnati, Cincinnati, OH, USA; 2 Biological Sciences Division, Pacific Northwest National Laboratory, Richland, WA, USA; 3 Department of Medicine, University of Washington School of Medicine, Seattle, WA, USA; 4 Department of Pathology, Microbiology and Immunology, Vanderbilt University School of Medicine, Nashville, TN, USA; 5 Division of Laboratory Sciences, National Center for Environmental Health, Centers for Disease Control and Prevention, Atlanta, GA, USA; 6 Division of Endocrinology, Department of Pediatrics, Cincinnati Children's Hospital Medical Center and University of Cincinnati, Cincinnati, OH, USA; 7 Department of Medicine, Vanderbilt University School of Medicine, Nashville, TN, USA

**Keywords:** high-density lipoprotein, HDL subfractions, lipoproteins, composition, mass spectrometry, differential ion mobility analysis, isotope-dilution MS/MS, ratchet model, size-exclusion chromatography, APOA1, apolipoprotein A-I, APOA2, apolipoprotein A-II, BS^3^, bis-(sulfosuccinimidyl) suberate, CETP, cholesteryl ester transfer protein, FPLC, fast protein liquid chromatography, GO, gene ontology, IAC, immunoaffinity chromatography, LpA-I, lipoproteins with APOA1 and no APOA2, LpA-I^L^, large subfraction of LpA-I, LpA-I^S^, small subfraction of LpA-I, LpA-I/A-II, lipoproteins with APOA1 and APOA2, PC, phosphatidylcholine, POPC, palmitoyl-2-oleoyl-*sn*-glycero-3-phosphocholine, SEC, size-exclusion chromatography, UC-HDL, HDL isolated by ultracentrifugation

## Abstract

Human high-density lipoproteins (HDLs) are a complex mixture of structurally related nanoparticles that perform distinct physiological functions. We previously showed that human HDL containing apolipoprotein A-I (APOA1) but not apolipoprotein A-II (APOA2), designated LpA-I, is composed primarily of two discretely sized populations. Here, we isolated these particles directly from human plasma by antibody affinity chromatography, separated them by high-resolution size-exclusion chromatography and performed a deep molecular characterization of each species. The large and small LpA-I populations were spherical with mean diameters of 109 Å and 91 Å, respectively. Unexpectedly, isotope dilution MS/MS with [^15^N]-APOA1 in concert with quantitation of particle concentration by calibrated ion mobility analysis demonstrated that the large particles contained fewer APOA1 molecules than the small particles; the stoichiometries were 3.0 and 3.7 molecules of APOA1 per particle, respectively. MS/MS experiments showed that the protein cargo of large LpA-I particles was more diverse. Human HDL and isolated particles containing both APOA1 and APOA2 exhibit a much wider range and variation of particle sizes than LpA-I, indicating that APOA2 is likely the major contributor to HDL size heterogeneity. We propose a ratchet model based on the trefoil structure of APOA1 whereby the helical cage maintaining particle structure has two “settings”—large and small—that accounts for these findings. This understanding of the determinants of HDL particle size and protein cargo distribution serves as a basis for determining the roles of HDL subpopulations in metabolism and disease states.

High-density lipoproteins (HDLs) are much more than the cholesterol shuttles they were thought of in the past (1). In addition to well-defined functions including lipid transport and anti-inflammatory processes, HDL is also associated with surprising roles in protease inhibition, vitamin transport, innate immune function, and even glucose control (2, 3, 4). These functions are undoubtedly mediated by a host of over 200 different proteins (http://homepages.uc.edu/∼davidswm/LDLproteome.html) (5) that have been found associated with human plasma HDL particles. Early on, it was generally thought that lipophilic apolipoproteins exchange randomly between all HDL particles. In a 1984 review, Eisenberg referred to HDL as a “temporary station for lipids and proteins” in which “the integrity of the HDL system appears to depend on and reflect the sum of the movements and modifications of its constituents” (6). Indeed this exchangeability is well documented for the two scaffold proteins APOA1 and APOA2 (7) as well as other apolipoproteins such as APOE (8) and the APOCs (9). However, work from numerous laboratories has shown that HDL particles can be separated into fractions that contain distinct complements of some of the lower-abundance HDL proteins. For example, HDL separated by density ultracentrifugation into five fractions showed remarkable disequilibrium with many proteins preferring the smallest, densest particles while others gravitated toward larger, lighter species (10). This has been further demonstrated using 2-D gel electrophoresis (11), gel filtration (12, 13), and ion exchange chromatography (12, 13). Recent studies have taken advantage of immunoaffinity chromatography to define and isolate HDL subfractions based on minor protein components (14) and have related these to incidence of cardiovascular disease (15) and diabetes (16) in large human studies. Thus, the term “HDL” actually refers to a family of particles with quite diverse compositions and functions, some related to cardiovascular disease, but others likely not. Nevertheless, relatively few studies have targeted individual HDL subspecies for comprehensive compositional analyses to better understand how the complement of minor HDL-associated proteins (which we like to call accessory proteins) defines their functionality. The biggest barrier has been the fact that HDL subspecies share numerous physicochemical properties that make them difficult to physically separate.

Cheung and Albers first took advantage of immunoaffinity chromatography (17) by targeting apolipoproteins A-I (APOA1) and A-II (APOA2), the two most abundant HDL proteins. Human plasma HDL was fractionated into populations that contained APOA1, but no APOA2 (LpA-I), and populations that contained both (LpA-I/A-II). The subfractions differed in size and content of a handful of accessory proteins that, at the time, were only identifiable by immunoblot. These have subsequently been studied extensively in terms of their rates of plasma clearance (18), levels and distribution in humans (19, 20), ability to promote cholesterol efflux from several cell types (21, 22, 23, 24), and ability to donate cholesterol in selective cholesterol uptake in the liver (25). However, the role of these subfractions in cardiovascular disease (CVD) remains unclear with some studies arguing for a linkage of one subtype or another in CVD (23, 26, 27) while others show no major relationships of either subfraction in CVD (28, 29).

We recently revisited these LpA-I and LpA-I/A-II subfractions and applied unbiased, bottom-up mass spectrometry to define their proteomic signatures (30). While many of the identified proteins were shared between the subfractions, the LpA-I population contained numerous unique proteins. Gene ontology analysis indicated that the LpA-I fraction was enriched in the proteins associated with the more “nontraditional” pathways of HDL metabolism such as inflammatory response, hemostasis, immunity, and protease inhibition. Visualizing these species with nondenaturing PAGE, we were struck by the fact that the LpA-I population presented as two relatively tight bands. This particle quantization was in stark contrast to similar analyses of either total HDL or LpA-I/A-II subfractions, which run as heterogeneous streaks or smears on native gels. Given the trademark heterogeneity of the HDL family, we reasoned that a detailed compositional study of these discreetly sized LpA-I particles may offer clues to the factors that govern HDL complexity. We used immunoaffinity chromatography to isolate the LpA-I subfraction from human plasma, which was then separated by high-resolution size-exclusion chromatography. The “pure” sized populations were extensively characterized in terms of protein and lipid composition, particle size, and morphology. The results indicate that HDL size, in large part, is dictated by resident APOA1 molecules.

## Materials and methods

### Isolation of lipoprotein subspecies

For the bulk of these studies, three normolipidemic human blood donors were used to characterize these LpA-I particles, a 52-year-old male, a 25-year-old male, and a 41-year-old female. All subjects were within normal BMI ranges with no outward metabolic health issues. After a 12 h fast, blood was collected into tubes containing 3.2% sodium citrate under an approved IRB protocol from the University of Cincinnati (this work abides by the Declaration of Helsinki principles). Where noted, anonymously donated, outdated human plasma from the Hoxworth Blood Center was also used for some particle preparations, but all compositional characterizations were performed on the freshly donated samples. Plasma was isolated by centrifugation at 1,000 *g* for 15 min at 4 °C and preserved in sucrose (5%, w/v) at –80 °C until ready for speciation studies. Plasma was thawed and lipoproteins containing APOA1 without APOA2 (LpA-I) were isolated using immunoaffinity chromatography (IAC) as previously described (30). Briefly, plasma was applied to an IAC column containing antibodies to APOA1 (Academy Biomedical) on a fast protein liquid chromatography (FPLC) system at 0.5 ml/min in phosphate buffered saline (PBS, 10 mM PBS, 140 mM NaCl, 0.01% EDTA, 0.01% azide, pH = 7.4). Bound lipoproteins were eluted in 3 M sodium thiocyanate (NaSCN) in PBS and immediately desalted using polyacrylamide 6,000 columns (Thermo Fisher Scientific). Eluent was concentrated and applied to an IAC column containing antibodies to APOA2 (Academy Biomedical) at 0.5 ml/min in PBS. Unbound particles containing no APOA2 were collected, concentrated, and analyzed by 4%–15% SDS PAGE to confirm the absence of APOA2. The LpA-I particles were subfractionated by using high-resolution gel filtration chromatography with four Superdex 200 10/300 columns (GE Healthcare) in tandem operating at a flow rate of 0.15 ml/min in PBS. The elution profile was monitored by UV light absorption at 280 nm and elution volumes corresponding to the two major peaks were determined using Peakfit v4.12 (Systat Software Inc); fractions 40–44 ml were pooled and designated as LpA-I Large (LpA-I^L^), and fractions 45–49 ml were pooled and designated as LpA-I Small (LpA-I^S^). The particles were concentrated, purged under N_2_, and stored at 4 °C until biochemical analysis, which was performed within 14 days of isolation.

### Particle size measurements

*Native PAGE:* 3 μg of total protein from each particle was visualized on an 8%–25% native PAGE separated on a GE Phast System. Protein was visualized by staining with Coomassie blue. *Negative stain EM*: The particles were stained using uranyl formate as previously described (31, 32) with modifications. Briefly, a formvar/carbon-coated transmission electron microscopy (TEM) grid was glow discharged and suspended on a 20 μl drop of sample (0.1 mg/ml) for 30 s. Excess sample was removed from the surface by blotting, the grid was washed twice with deionized water and stained with 0.75% uranyl formate (pH = 4.5). Excess stain was removed by blotting and the grid was air dried in darkness. Grids were imaged on an FEI Tecnai T12 transmission electron microscope operating at 100 kV. Micrographs were collected at 150,000× magnification with a pixel size of 3.9 Å/pixel using an AMT XR-41 CCD camera and imaging software. For quantitation of particle size distribution, the diameters of ten particles from each of ten micrographs, selected at random, were measured (100 total diameter measurements for each sample) using FIJI (ImageJ) image analysis software. *Differential mobility analysis:* Molar particle concentrations and size of the particles were directly measured using calibrated differential ion mobility analysis (IMA) on a differential mobility analyzer (TSI Inc., Minnesota) (33). The measured profiles were quantified after fitting the raw data with the use of Fityk by an unsupervised fitting algorithm (34). Peak areas were externally calibrated with a five-point calibration curve using a protein standard to determine the molar concentration of the particles (33). Intraday and interday imprecision of the method was <10%.

### Particle composition

Phospholipid, total cholesterol, free cholesterol, and triglyceride content of particles were determined enzymatically using the Phospholipids C Kit (Wako Cat# 997-01801), Triglyceride Reagent (Pointe Scientific Cat# T7532), and the Amplex Red Total and Free Cholesterol Assay kit (Thermo Fisher Scientific Cat# A12216). Cholesteryl ester was determined from the difference between total and free cholesterol multiplied by 1.67 to account for the mass of the acyl chain. Protein was determined by the Markwell Lowry assay (35).

### Lipid composition by mass spectrometry

The concentration of the main phospholipids (PL), as a total of phosphatidylinositols (PI), phosphatidylcholines (PC), phosphatidylethanolamines (PE), sphingomyelins (SM), and lyso-phosphatidylcholines (LPC), was determined by HILIC chromatography separation and PL species targeted MS/MS detection (36). A similar one-pot extraction protocol was used as for the nonpolar lipids, but for the extraction/reconstitution of the dried extracts, a 200-μL mix of nonane, isopropanol, and water was used (55:43:2 proportions). From the supernatant in each well, 5 μl was injected into the Acquity UHPLC system (Waters, USA) equipped with a Kinetex HILIC 100 Å pore, 2.1 × 100 mm, 1.7 μm particle column. The separation was a gradient elution with a flow rate of 0.7 ml/min. Mobile phase A was 99:1 acetonitrile:isopropanol. Mobile phase B was 2.5 mM aqueous ammonium acetate in 1:1 acetonitrile:water. The gradient was from 20% B to 50% B over 1.0 min, held for 0.3 min, 50%–100% B over 0.1 min, held for 0.8 min, returned to 80% A and 20% B over 0.01 min, and held for 0.79 min. The total run time was 3.0 min. A 6500 Qtrap (Sciex, Framingham, MA) was operated in MRM scanning mode with the TurboSpray IonDrive source (ESI). PI species were monitored in negative ion mode, and the remaining classes, PC, PE, SM, and LPC were monitored in positive ion mode. Only typical fatty acid analogs were monitored that are present at the highest concentration in human plasma, 15 for PI, 19 for PC, 19 for PE, and 8 for LPC, using species-specific total molecular ions as precursors and generic head group product ions. For the quantification of the total of lipid classes (PI, PC, SM, PE, LPC), the dilution series of a value-assigned plasma pool was used. Individual lipid species were quantified based on the native/labeled signal intensity ratio multiplied by the concentration of the labeled analog value assigned by the vendor. Lipid species had to show up in all three technical replicates of either LpA-I^L^ or LpA-I^S^ for inclusion in the analysis. Data was imported into Perseus (37) for final analysis.

### Protein composition by mass spectrometry

55 pmol of the LpA-I particles, determined by calibrated IMA, was diluted with 1% sodium deoxycholate (SDC) in 200 mM ammonium bicarbonate to 0.5% SDC final concentration and spiked with 5 μg of ^15^N-APOA1 standard (^15^N-APOA1 concentration accurately determined by amino acid analysis) for approximately equimolar concentration of the endogenous and the ^15^N-APOA1 internal standard. Samples were then reduced with dithiothreitol, alkylated with iodoacetamide, and digested with trypsin (1:10, w/w; Promega, WI) overnight at 37 °C. Trypsin digestion was quenched by acidification to pH 2.5 (with 10% formic acid) and incubated for 10 min to precipitate SDC. SDC precipitate was separated by centrifugation and the supernatant was collected and analyzed by LCMS. The digests were desalted on a C18 trapping column (Reprosil-Pur 120 C18-AQ, 5 μm, 0.1 × 40 mm, Dr Maisch HPLC GmbH, Ammerbuch-Entringen, Germany) (trapping flow rate 4 μl/min), separated on a capillary analytical column (Reprosil-Pur 120 C18-AQ, 5 μm, 250 × 0.075 mm, Dr Maisch HPLC GmbH) with a 30 min linear gradient of acetonitrile, 0.1% formic acid (7%–35%) in 0.1% formic acid in water at a flow rate of 0.4 μl/min using a nanoAquity UPLC (Waters, MA), and analyzed in a Thermo Orbitrap Fusion Lumos (Thermo Fisher) mass spectrometer with electrospray ionization. The instrument was operated in Parallel Reaction Monitoring (PRM) mode with selection in the quadrupole analyzed with a window of 1.6 m/z and HCD fragmentation with normalized collision energy NCE = 30. Production ions spectrum was acquired in the orbitrap analyzed at resolution of 15,000 and maximum fill time of 22 ms. Spectra were acquired and processed using Xcalibur (ThermoScientific, v 4.4.16.14).

Data-dependent acquisition was performed on three independently isolated samples each of LpA-I^L^ and LpA-I^S^ particles (from the same donor, isolated over about 1 year). Mascot generic files were generated from the raw data using MSConvert (v 3.0) (38) and experimental peptide spectra were matched to the UniProtKP/Swiss-prot protein knowledgebase for Homo Sapiens (version: release 2017_11, date: 12-20-2017, entries: 20,319) using the Mascot search engine (v 2.2.07). Trypsin was used to generate peptides and data were constrained to allow for a maximum of three missed cleavages, fixed modification of carbamidomethylation, and a variable modification of oxidation with a peptide tolerance of 0.15 Da and MS/MS tolerance of 0.15 Da. Scaffold (v4.3.4) was employed for MS/MS peptide validation using X! Tandem (2010.12.01.1, subset of 460 entries). Identified proteins and peptides were constrained to 99.9% and 95.0% probability, respectively, and protein inclusion required identification of at least three unique peptides and a given protein had to show up in the same fraction (either LpA-I^L^ or LpA-I^S^) across all three independent isolations. A final list of 94 proteins were selected and integrated into a FASTA file. Data-independent acquisition was performed on four independently isolated samples of LpA-I^L^ and LpA-I^S^ particles (also from the same donor, isolated over about 1 year). Raw data files and the custom FASTA file generated above were used to perform label-free quantification (LFQ) using MaxQuant (39). Samples were further processed by exporting the protein LFQ data into Perseus (37) for final analysis.

### Chemical cross-linking

Particles were cross-linked using the homobifunctional cross-linker Bis(sulfosuccinimidyl)suberate (BS^3^). Particles were cross-linked at a protein concentration of 1 mg/ml in PBS. We estimated APOA1 to account for approximately 80% of the total protein in each prep and BS^3^ was added to respective particles at a molar ratio of 50:1 BS^3^:APOA1. The reaction was carried out at 4 °C for 16 h. Cross-linked particles were visualized by running 8 μg on a 4%–15% SDS-PAGE and staining with Coomassie blue.

### Particle dimension and volume analysis

The particle diameters and lipoprotein constituent measurements accumulated in this study were used to perform a geometric analysis of the LpA-I^L^ and LpA-I^S^ particles. The mass data from the enzymatic compositional assays were used to calculate molar concentrations using 28,016 Da for APOA1, 758 Da for phospholipid, 387 Da for free cholesterol, 625 Da for cholesteryl ester, and 885 Da for triglyceride. Fraction of protein identified as APOA1 was taken from the quantitative MS data (75.5% for LpA-I^L^, 85% for LpA-I^S^). Neutral lipid core diameters and volumes were calculated by subtracting 20.5 Å from the experimentally derived particle radii to account for the thickness of the surface monolayer (40). Molecular volumes of 1,179 and 1,575 Å^3^ were used for cholesteryl ester and triglyceride, respectively (41) Surface area calculations used values of 15.5 Å^2^ per a.a. for fully compressed APOA1 (42) and 65 Å^2^ for phospholipids (43). Free cholesterol was not added as it is thought to not contribute substantially to net surface area of lipoproteins (40).

### Statistics

All data are presented as averages ± standard deviation (SD). Lipidomics and proteomics data were analyzed by multiple sampling ANOVA with a permutation-based FDR threshold of 5% (n = 250 randomizations) to generate a q-value. False discovery rate–adjusted significance levels were computed for pairwise comparisons: statistical significance was q < 0.05. Compositional analyses from enzymatic measurements were evaluated using one-way ANOVA and statistical significance was *P* < 0.05 with a Tukey's honestly significant difference test.

## Results

### LpA-I subspecies isolation and sizing

**Fig. 1:**
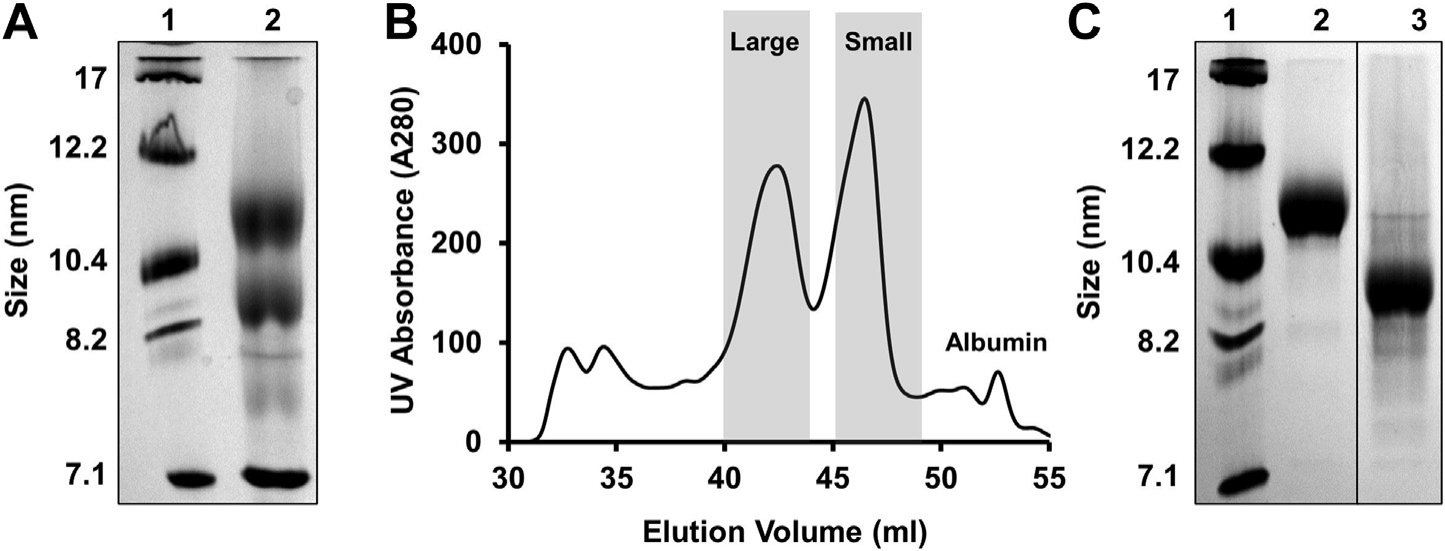
Isolation of LpA-I^L^ and LpA-I^S^ from human plasma. Plasma LpA-I particles were isolated using immunoaffinity chromatography and applied to four Superdex 200 columns in series. A: Native PAGGE analysis of the plasma LpA-I subfraction. Lane 1: MW standards, (lane 2) Plasma LpA-I particles. B: Elution profile of the plasma LpA-I subfraction. Elution volumes collected and pooled for LpA-I^L^ and LpA-I^S^ particles are shaded in gray. C: Native PAGGE analysis of LpA-I^L^ and LpA-I^S^ subfractions. Lane 1: MW standards, (lane 2) LpA-I^L^ particles, (lane 3) LpA-I^S^ particles. Both gels were stained with Coomassie blue. Subfractions are from plasma from the same individual and same isolation (52-year-old male). The line in (C) represents a break in the same gel where samples irrelevant to the current study were removed.

Native PAGGE analysis of HDL isolated by density ultracentrifugation from different humans demonstrates wide variations in the size distribution of the particles (8–12 nm in diameter) and intensity of protein staining (supplemental Fig. S1A), reflecting the well-known size and compositional heterogeneity of HDL. We (30) and others (41, 44) have shown that when human UC-isolated HDL is separated into its LpA-I and LpA-I/A-II components by immunoaffinity chromatography, the LpA-I fraction resolves as two remarkably discrete bands by native PAGGE while LpA-I/A-II remains as an unfocused smear like total HDL. Because isolation of HDL by UC significantly impacts particle composition (45), we first set out to determine whether these populations exist to the same extent in the LpA-I fraction isolated directly from human plasma without an intervening centrifugation step. Figure 1A (lane 2) shows a native PAGGE analysis of the LpA-I fraction from a 52-year-old, normolipidemic male donor directly after immunoaffinity isolation. Three major bands are apparent. The lowest was identified as residual human serum albumin, while the two larger bands correspond to the large (LpA-I^L^) and small (LpA-I^S^) subfractions previously observed in UC-isolated HDL. As shown in supplemental Fig. S1B, our standard setup of high-resolution size-exclusion chromatography with three Superdex columns in series (12) failed to provide enough separation of these populations for independent characterization. By integrating a fourth column in series and reducing the flow rate, we obtained the elution profile for plasma LpA-I in Fig. 1B. The bulk of the protein eluted in two peaks corresponding to the native PAGGE bands. Additional protein peaks were apparent nearer the column void volume. These likely reflect APOA1-containing VLDL and LDL particles captured by the antibodies. A peak shape analysis using Peakfit (Systat) indicated that the shaded fractions would result in negligible overlap of the LpA-I^L^ and LpA-I^S^ particles. Indeed, native PAGGE (Fig. 1C) showed excellent segregation of the two populations after pooling. These species were not unique to our initial blood donor. Similar separations run on three separate anonymous plasma lots from the Hoxworth Blood Center resulted in similar LpA-I^L^ and LpA-I^S^ particles with highly consistent diameters by gel filtration (supplemental Fig. S2A) and native PAGGE (supplemental Fig. S2B). This indicates these particles are stable in fresh or refrigerated (for ∼2 weeks) plasma.

To get an idea of how age and sex affect the size and composition of LpA-I^L^ and LpA-I^S^, we performed LpA-I isolations on three fresh plasma donors. This included the 52-year-old male described above, a 41-year-old female, and a younger male (25-year-old). Given the labor-intensive nature of the purification, we performed compositional and sizing analyses on particles from all three donors. For more detailed analyses such as the proteomics and lipidomics, we utilized three independent isolations from the 52-year-old donor.

Native gel analysis showed the LpA-I^L^ particles exhibited hydrodynamic diameters of 106–108 Å, whereas LpA-I^S^ were 81–82 Å (Table 1) across all three subjects. This indicates that size and presence of LpA-I^L^ and LpA-I^S^ subfractions were highly consistent across individuals of different age and sex. However, the relative concentration (i.e., peak heights) of the LpA-I^L^ did show variability between the individuals (not shown).

**Table 1:** Particle size determinations of LpA-I^L^ and LpA-I^S^ subfractions

Particle	Subject	n	Native PAGE (Å)	EM (Å)	Calibrated IMA (Å)
LpA-I^L^	52 years old, male	3	108 ± 2	107 ± 4	109 ± 1
	41 years old, female	1	106	ND	108
	25 years old, male	1	107	ND	109
LpA-I^S^	52 years old, male	3	82 ± 1	91 ± 1	91 ± 1
	41 years old, female	1	81	ND	90
	25 years old, male	1	81	ND	90

Particle size determinations were performed using three independent methods. Values represent the mean (± 1 SD) particle diameters for three independent isolations across the same donor (52 years old male). A single prep and size measurement were generated from each of the other two donors.

**Fig. 2:**
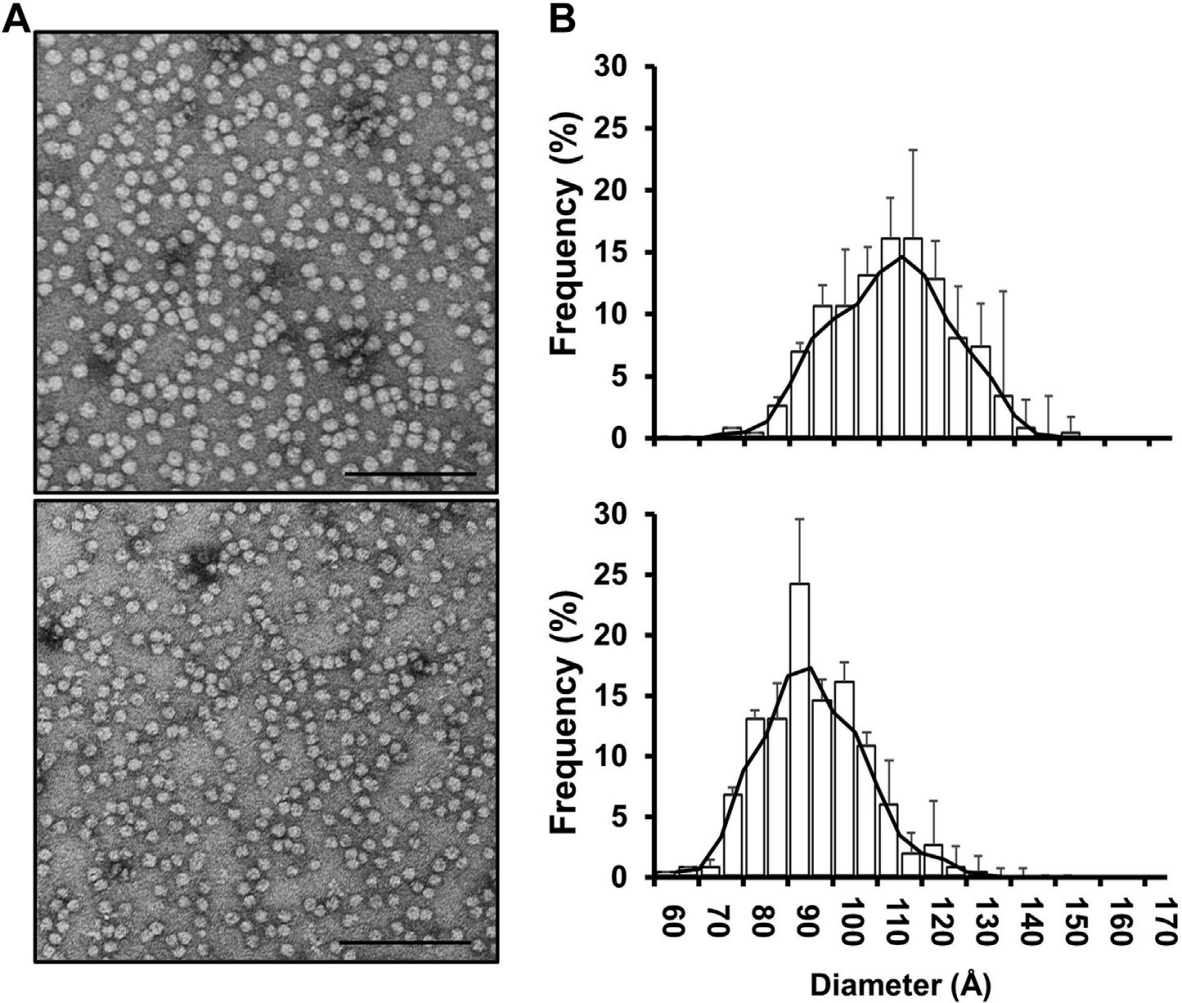
Negative stain EM of LpA-I^L^ and LpA-I^S^ particles. Human plasma LpA-I^L^ and LpA-I^S^ particles were stained and visualized using an FEI Tecnai T12 transmission electron microscope. A: Representative micrographs of LpA-I^L^ (top) and LpA-I^S^ (bottom) particles. Lines on the images represent 1,000 Å. B: Histogram of LpA-I^L^ (top) and LpA-I^S^ (bottom) particle diameters. Bars represent mean of observed diameter frequency (± 1 SD) from three independent isolations from the same individual (52-year-old male).

**Fig. 3:**
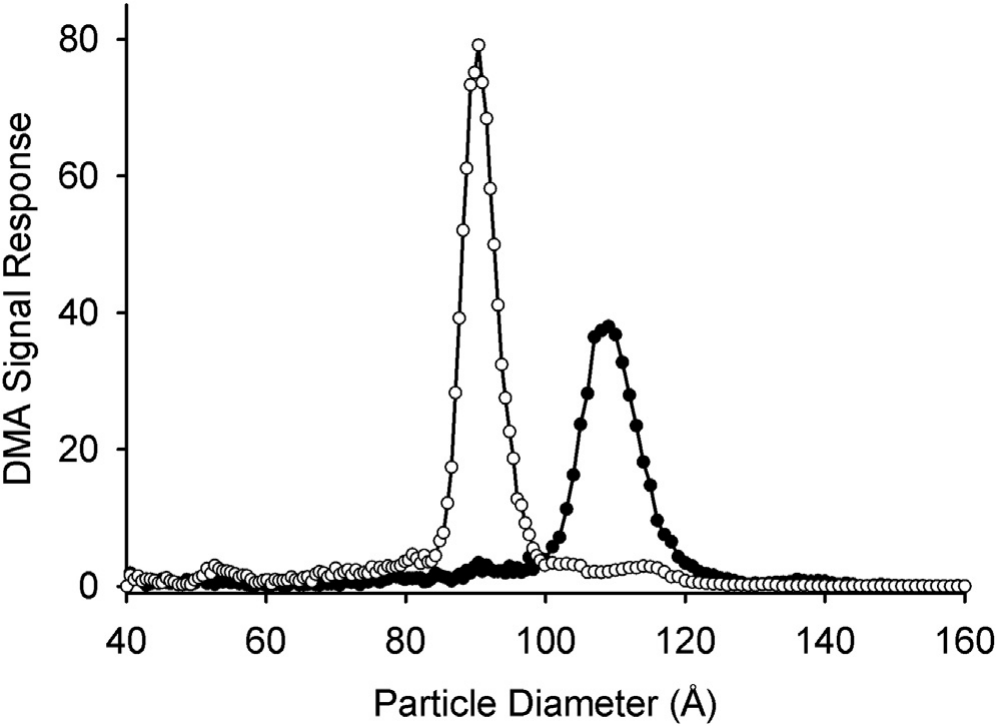
Quantification of particle size by calibrated IMA. LpA-I^L^ and LpA-I^S^ particles were analyzed by calibrated ion mobility analysis (Materials and Methods). Representative profiles for LpA-I^L^ (black circles) and LpA-I^S^ (white circles) are shown from one of three independent preparations from a single donor (52-year-old male).

The particles isolated from the 52-year-old male were also assessed by negative stain EM. Both populations appeared spherical (Fig. 2A) with generally Gaussian-like size distributions (Fig. 2B). The average diameters calculated for LpA-I^L^ and LpA-I^S^ were 107 Å, and 91 Å, respectively (Table 1). Given the discrepancy in size of LpA-I^S^ between native PAGGE and EM, we opted to apply an additional orthogonal method to measure particle diameter: ion mobility analysis (IMA) (33). Originally developed for analyzing aerosol particles, this method measures the velocity of lipoproteins as they traverse an electric field with a known electrical potential. The diameter and number of particles registered by the detector are calculated using first principles; the method accurately determines the sizes and concentrations of reconstituted HDL, gold nanoparticles, and proteins with a wide range of MWs and isoelectric points (46). Figure 3 shows representative calibrated IMA profiles of the two isolated LpA-I species from the 52-year-old male donor. Consistent with the native PAGE and SEC results, the populations exhibited minimal overlap. The measured diameters, 109 Å for LpA-I^L^ and 91 Å for LpA-I^S^, were in remarkable agreement with the EM results (Table 1). The particles from the younger male and female donor exhibited highly similar diameters by calibrated IMA (supplemental Fig. S3 and Table 1). This further confirms the striking consistency in LpA-I particle size among different individuals. The native PAGGE appeared to underestimate the particle size in the case of LpA-I^S^ in all cases. The reason for this is not clear, but could result from variations in particle shape or failure of the electrophoresis to reach a true equilibrium state in the time allowed.

### Lipid composition

A general analysis of the composition of the particles was performed using enzymatic assays. Table 2 summarizes the distribution of the major compositional components from three independent preparations from the 52-year-old male donor and single preparations from the younger male and female subjects. On average, LpA-I^L^ contained about 44% protein and 56% lipid (combined PL, FC, CE, TG), whereas LpA-I^S^ was more enriched in protein at ∼52%. The protein to lipid weight distributions for LpA-I^L^ were comparable to those measured for UC isolated HDL_2_ (∼41% protein) while the LpA-I^S^ were slightly more enriched in lipid than typical for UC isolated HDL_3_ (∼55% protein) (47). Phospholipid was the primary lipid class on both subfractions followed closely by cholesteryl ester. Free cholesterol and triglycerides were minor components. Nonetheless, the LpA-I^L^ particles were consistently enriched in FC as a ratio to total PL. The relative composition of the neutral lipid ester core was not different between the two species as the TG/CE ratio was similar between the two subfractions.

**Table 2:** Chemical composition of LpA-I^L^ and LpA-I^S^ subfractions

Particle	Subject^a^	n^b^	Pro (%)^c^	PL (%)	FC (%)	CE (%)	TG (%)
LpA-I^L^	52 years old, male	3	39 ± 1.8∗	30 ± 1.8	3.9 ± 0.2^#^	25 ± 0.5^&^	2.5 ± 0.5
	41 years old, female	1	51	26	3	19	2
	25 years old, male	1	43	28	3	21	6
	Average		44 ± 6	28 ± 2	3 ± 1	22 ± 3	3 ± 2
LpA-I^S^	52 years old, male	3	48.0 ± 1.6∗	26 ± 1.4	2.2 ± 0.2^#^	22 ± 0.7^&^	2.0 ± 0.3
	41 years old, female	1	58	24	1	15	2
	25 years old, male	1	51	26	2	17	4
	Average		52 ± 5	25 ± 1	2 ± 1	18 ± 3	3 ± 2

a Three fasted, normolipidemic subjects of varying age and sex were analyzed for these compositional analyses. The particle separation protocol was initiated immediately after blood draw in each case.

b One donor was used to generate three independent preparations of particles over a span of about 6 months. Values from that individual are reported ± 1 sample standard deviation. Values with the same symbol (∗, #, or &) were significantly different between particle size species by *t* test at *P*< 0.05. *P*-values for the Pro, FC, and CE comparisons were 0.0027, 0.0002, and 0.0027, respectively. The other two donors provided a single preparation each and are thus reported without statistics.

c Protein and lipid components of each particle were measured and expressed as percentage (wt/wt) of the total mass of all components combined. Average is across all subjects (equally weighted) is reported ± 1 sample standard deviation.

**Fig. 4:**
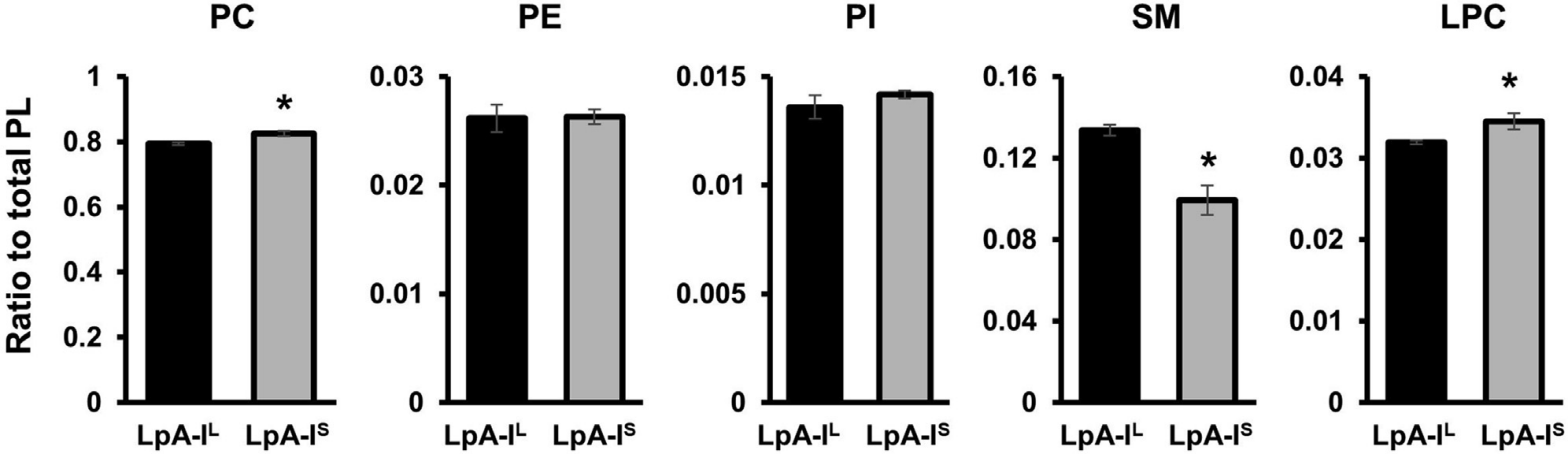
Lipid subclasses identified in LpA-I^L^ and LpA-I^S^ by quantitative mass spectrometry. Equal total protein amounts of each subclass were analyzed by MS as described in Materials and Methods. Selected mass ratios for various lipids are shown. Error bars represent one standard deviation from three technical replicates of particles from the 52-year-old male donor. Asterisk denotes statistically significant differences (see Materials and Methods).

Given the labor-intensive protocol for isolating these particles, we elected to move forward with more detailed analyses on the triplicate particle isolations from the 52-year-old male donor. We performed a detailed lipidomics analysis by LC-MS to investigate differences in enrichment of individual lipid species between the two subfractions. Ratios of individual phospholipid classes to total phospholipid were used as a measure of enrichment. Most of the phospholipid species were similar between LpA-I^S^ and LpA-I^L^ (Fig. 4); however, the LpA-I^S^ fraction showed a small enrichment in both LPC and PC. The most striking observation was the enrichment of SM in the LpA-I^L^ subfraction. Indeed, LpA-I^S^ exhibited decreases in all but two of the SM species compared with the LpA-I^L^; values for all detected lipid species across the three replicates and LpA-I^L^ and LpA-I^S^ subfractions can be found in supplemental Table S1.

### Protein cargo

**Fig. 5:**
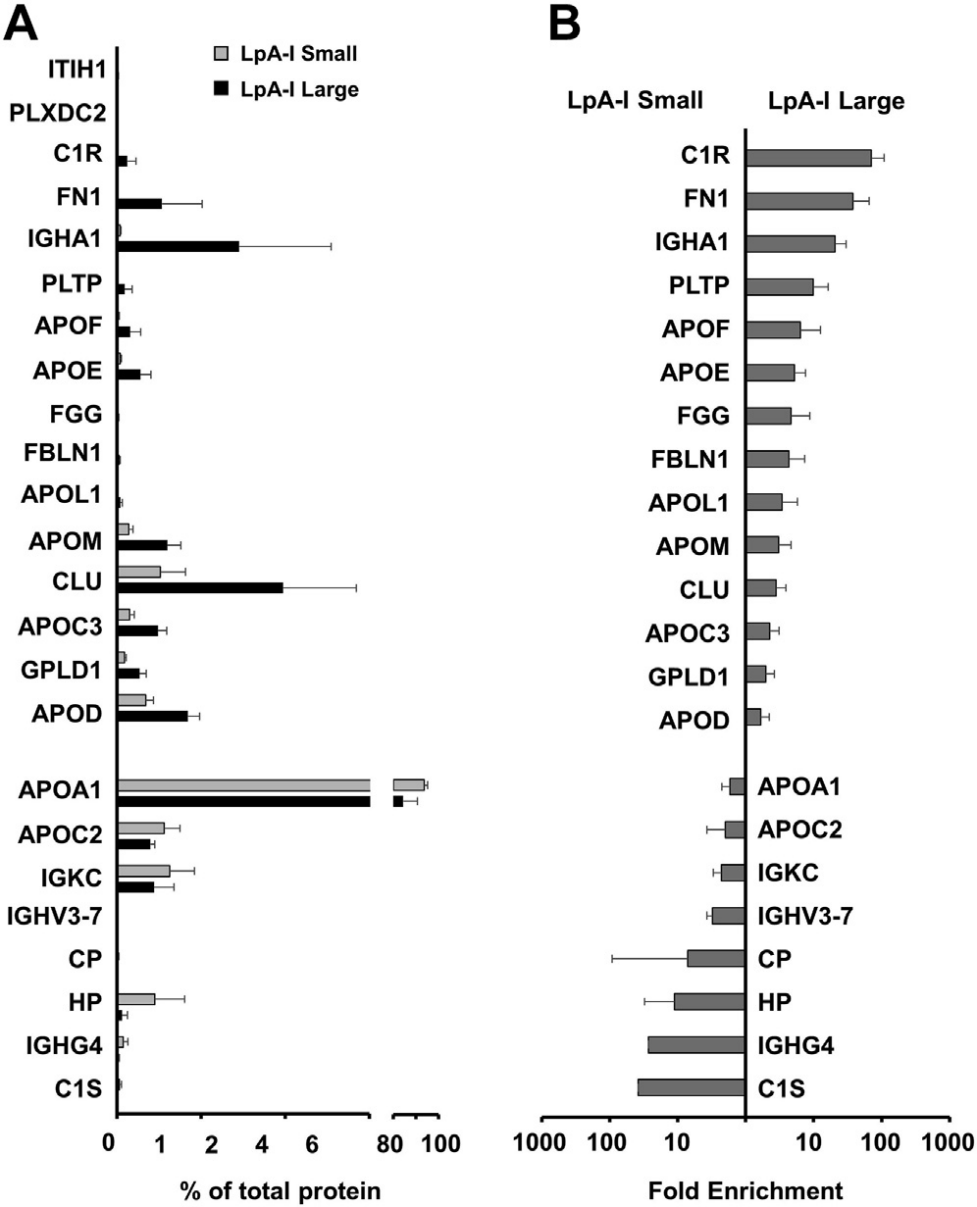
Proteomic differences between LpA-I^L^ and LpA-I^S^. Proteins were identified using LC-MS as described. A: Protein abundance of significantly different proteins (q < 0.05) between the two particle populations expressed as a percentage of total protein abundance. Protein abundance was determined using label-free quantification with MaxQuant. B: Fold enrichment of proteins in LpA-I^S^ (left) and LpA-I^L^ (right) that were statistically significant different between the two populations (q < 0.05). Bars represent the mean (± 1 SD) of particles from four independent isolations from the 52-year-old male donor.

We next looked at the protein composition of the particles by semiquantitative MS/MS. We initially identified 94 proteins in all independent particle preparations that met the conditions laid out in Materials and Methods. MaxQuant analysis quantified 84 of those; after removal of common contaminants such as keratins, 55 proteins remained that were detected in at least three out of four samples (supplemental Tables S2 and S3; the relative abundances for proteins in the LpA-I^L^ and LpA-I^S^ subfractions). All proteins have been previously identified in HDL preparations by various techniques (http://homepages.uc.edu/∼davidswm/LDLproteome.html). Importantly, APOA2 was barely detectable, indicating that the immuno-affinity separations faithfully isolated LpA-I species only. In total, we identified 24 proteins that were statistically different in abundance between the LpA-I^L^ and LpA-I^S^ particles (Fig. 5A) in this subject. All but two proteins were found in both subfractions with ITIH1 and PLXDC2 found exclusively in the LpA-I^L^ subfraction.

As expected, APOA1 accounted for the majority of the protein found in both size species with abundances of 76% and 85% in the LpA-I^L^ and LpA-I^S^, respectively. The next most abundant protein in the LpA-I^L^ particles was clusterin (APOJ ∼4%) followed by PON1 and IGHA1. The second most abundant protein in LpA-I^S^ particles was APOC1 followed by PON1 and IGHG2. Figure 5B shows the relative enrichment of each protein in LpA-I^L^ and LpA-I^S^, irrespective of abundance (proteins enriched in LpA-I^S^ are on the left vs. LpA-I^L^ on the right). The complement component C1s was highly enriched in LpA-I^S^ as was haptoglobin, ceruloplasmin, APOC2, and interestingly, APOA1. The LpA-I^L^ species appeared to exhibit the highest degree of proteomic diversity with enrichments in some 14 proteins. LpA-I^L^ was enriched in many of the classic “apos” that one typically associates with HDL including APOF, APOE, APOL1, APOM, clusterin, and APOC3. We went on to perform a gene ontology analysis to determine whether the proteins between subfractions partition into different functional pathways. Of the pathways investigated, we found the LpA-I^S^ subfraction appeared somewhat enriched in proteins important for proteolysis while the LpA-I^L^ favored proteins involved in apoptosis (supplemental Table S4). Protein assignments to the gene ontology functions can be found in supplemental Table S5.

### Scaffold protein stoichiometry

**Fig. 6:**
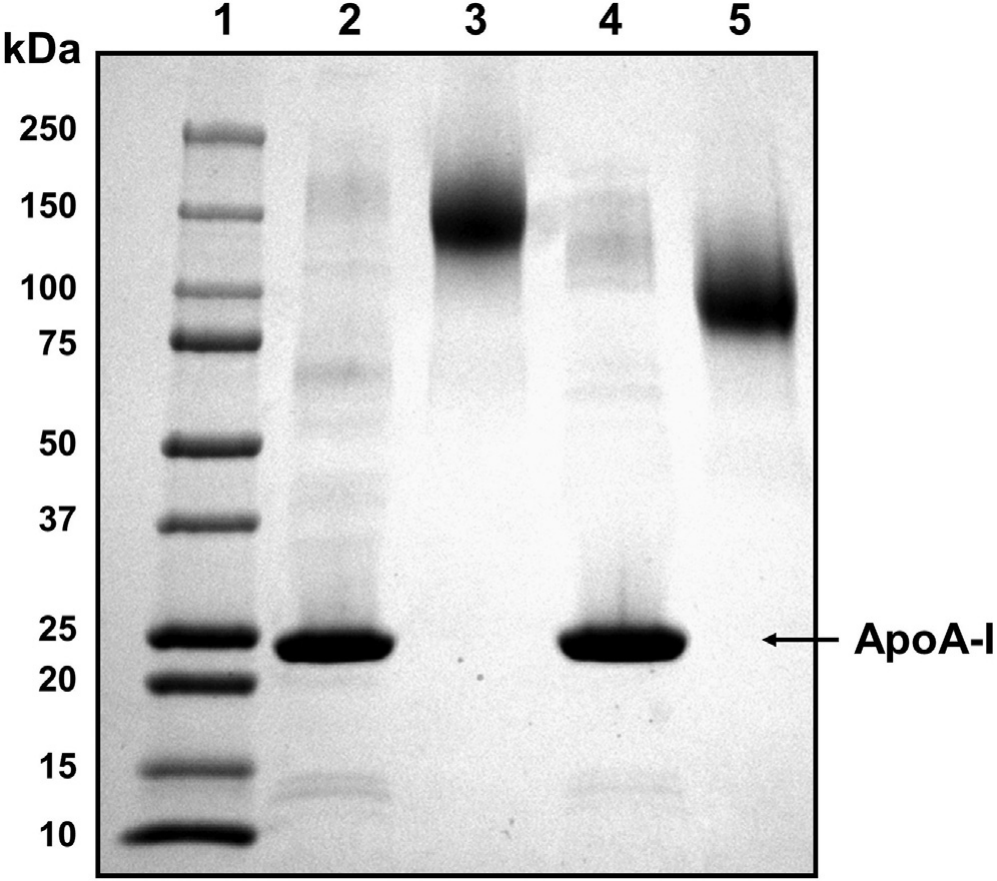
Chemical cross-linking of LpA-I^L^ and LpA-I^S^ proteins. Particles (8 μg protein) from the 52-year-old male donor were analyzed by SDS PAGGE on a 4%–15% gradient gel. Lane 1: MW ladder, (lane 2) LpA-I^L^ no cross-linker, (lane 3) LpA-I^L^ cross-linked with BS^3^ for 16 h at 4 °C, (lane 4) LpA-I^S^ no cross-linker, (lane 5) LpA-I^S^ cross-linked with BS^3^. The gel was stained with Coomassie blue.

To begin to understand the structure of the LpA-I^L^ and LpA-I^S^ populations, we determined the number of APOA1 molecules per particle. First, we chemically cross-linked LpA-I^L^ and LpA-I^S^ with a soluble homobifunctional cross-linking agent (BS^3^). SDS-PAGGE analysis of these particles before cross-linking demonstrated that APOA1 was the dominant protein species with additional protein bands evident (Fig. 6), consistent with the proteomics results above. LpA-I^S^ also contained predominately APOA1, but the minor protein constituents exhibited a different banding pattern vs. LpA-I^L^. Upon cross-linking, all the protein bands for LpA-I^L^ coalesced into a single band with a calculated MW of about 142 kDa. This indicated a near quantitative interconnection of all proteins on the particle surface. For LpA-I^S^, the corresponding band migrated faster with an apparent MW of ∼102 kDa. We have previously used this cross-linking analysis to roughly estimate the number of APOA1's per particle by assuming that APOA1 is the dominant species present on these particles (48). The size of the cross-linked product for LpA-I^L^ suggests a maximum of five molecules of APOA1 per particle while that for LpA-I^S^ suggests about 3.6 molecules. However, the disappearance of all minor bands upon cross-linking indicates that these proteins must also contribute to the final aggregate, but to an unknown extent. Therefore, we set out to derive a more accurate way to estimate the number of APOA1 molecules per particle.

In addition to measuring particle size, calibrated IMA analysis also quantifies the molar concentration of individual particles (33). This provides a critical denominator for measured APOA1 concentrations for determining particle stoichiometry. We split the respective subfractions into two aliquots. The first was used to determine particle number by calibrated IMA. The second was used to quantify APOA1 *via* isotope dilution MS/MS by comparing the ratio of the endogenous APOA1 peptides with those derived from [^15^N]-labeled APOA1 of known concentration, as determined by amino acid analysis (49). The molar concentrations of APOA1 were then used to calculate the number of APOA1's per particle (Table 3, two independent particle isolations from the 52-year-old male donor). Importantly, we calibrated our measurements with a positive control of reconstituted HDL particles with a well-established stoichiometry of two molecules of APOA1 per particle ((50) and supplemental Fig. S4). To our surprise, the results indicated that there were three molecules of APOA1 per particle for LpA-I^L^ and about 3.7 molecules for LpA-I^S^. The overestimation of the size of the LpA-I^L^ protein component from cross-linking/SDS-PAGE analysis (5 APOA1/particle) compared with the APOA1 stoichiometry (3 APOA1/particle) indicates that the accessory proteins carried on these particles contribute significantly to their surface area. For LpA-I^S^, the cross-linking suggests about 3.6 APOA1/particle, in excellent agreement with the stoichiometry measurement (3.7 APOA1/particle).

**Table 3:** APOA1 stoichiometry of LpA-I^L^ and LpA-I^S^ particles

Particle	[Particles] (μM)	[APOA1] (μM)	APOA1/Particle Experimental	APOA1/Particle Normalized
LpA-I^L^	9.9 ± 1.1	35 ± 2.8	3.5 ± 0.1	3.1 ± 0.1
LpA-I^S^	9.2 ± 0.6	38 ± 0.5	4.2 ± 0.3	3.7 ± 0.3
rHDL	21 ± 1.8	47 ± 4.8	2.3 ± 0.1	2.0 ± 0.0

Stoichiometry was determined using calibrated IMA and isotope dilution MS/MS with [^15^N]-APOA1 as internal standard. Analyses were performed on independent preparations of the LpA-I subfractions from the 52-year-old donor with accompanying 96 Å rHDL standards for each preparation. Each analysis contained three technical replicates. Data was normalized to rHDL standards (two molecules of APOA1 per particle as determined by cross-linking and SDS-PAGE). Values represents the mean (± SD, n = 6) for two separate experiments.

## Discussion

We were intrigued by the discrete size populations in the LpA-I fraction compared with the extreme heterogeneity of affinity isolated LpA-I/A-II or total HDL. Our investigations indicate that these particles are remarkably consistent in size in males between 25 and 52 years old and in females. In fact, in every plasma sample we have looked at, LpA-I^L^ and LpA-I^S^ appear and have remarkably similar diameters. Thus, these particles are relatively common, at least in normolipidemic humans. As LpA-I represent about one-third of plasma HDL (51), we estimate that LpA-I^L^ and LpA-I^S^ could account for 9%–12% and 15%–20% of total plasma APOA1, respectively, depending on the individual. To better understand the structure and function of HDL, we report the most extensive molecular characterization of individual LpA-I size subfractions to date.

### APOA1's role in HDL particle size in vivo

Given our previous observations that the LpA-I particles are more diverse with respect to minor proteins than LpA-I/A-II particles (52), one might think that the LpA-I subfraction should be a major driver of overall HDL size heterogeneity. However, our results indicate that accessory surface proteins are not the major factor determining LpA-I particle size. We detected up to 55 proteins across the LpA-I^L^ and LpA-I^S^ subfractions, few of which can be present on the same particle at the same time, yet each class of the particles remained within a remarkably tight size range. One might also predict that a principal driver of HDL particle diameter is the size of its neutral lipid core, created by the actions of HDL remodeling factors such as LCAT and CETP, with the surface lipids and proteins coating whatever size droplet is present. This certainly appears to be the case for LpA-I/A-II particles, which can adopt any diameter between 7 and 11 nm. However, the fact that LpA-I particles are focused into two discreet size populations indicates that the neutral lipid core size is constrained by some other factor. It is difficult to imagine that the activities of LCAT or CETP could be so precisely regulated in LpA-I to create only two discrete sizes of particles. It seems more reasonable that some structural constraint unique to LpA-I can limit the size of the neutral lipid core to two “choices,” large and small.

The structure of the major HDL protein APOA1 may offer a plausible explanation. We suggest that the amphipathic helical cage structure created by APOA1 creates vessels that can be filled with specific amounts of lipid. In the case of human LpA-I particles, this cage structure seems to have two “settings”—small and large. This is consistent with many years of size studies of APOA1-containing reconstituted HDL particles. Jonas and colleagues convincingly showed that APOA1 and POPC form discs with discrete sizes of 78, 85 and 96 Å—with nothing in between—despite each containing only two molecules of APOA1 (53). The changes in diameter were proposed to result from conformational changes in APOA1 that expanded or contracted in specific steps to encapsulate a patch of phospholipid bilayer. In spherical human LpA-I^L^, the 109 Å diameter particle may arise from all three of its APOA1 molecules maximally stretching across the particle surface. We have previously proposed the trefoil model for APOA1's structure in human HDL (48). It holds that three molecules of APOA1 encapsulate a sphere of lipid by bending into wedge-shaped structures that interdigitate to create a spherical cage. Interestingly, the three APOA1 molecules in this arrangement maintain the same intermolecular helical interactions exhibited by two APOA1 molecules in a discoidal double belt (54). These same interactions were present in two X-ray crystal structures of oligomerized truncation mutants of lipid-free APOA1 (55, 56). Like in the discs, we propose that the trefoil scaffold can undergo a ratcheted conformational change to transition from a small to a large sphere (and vice versa) when available lipid warrants. In other words, LpA-I^S^ and LpA-I^L^ particles utilize the same APOA1 scaffold, but in different states of extension. Indeed, our original modeling estimated that a fully extended APOA1 trefoil could stabilize a particle with a maximal diameter of ∼110 Å, in excellent agreement with the experimentally measured diameter of human plasma LpA-I^L^. Further studies will be required to determine the metabolic factors that contribute to the lipid available to produce these particles and whether/how they can interconvert.

A previous study by Segrest *et al.* (41) characterized LpA-I particles isolated from UC HDL and estimated the number of APOA1 molecules per particle based on volumetric calculations. The particle corresponding to LpA-I^L^ (called HDL[7]) was estimated to have four APOA1s per particle. LpA-I^S^ (called HDL[4]) was predicted to have three molecules (note: Segrest *et al.* also identified a third, smaller LpA-I species (called HDL[1]) that was predicted to have two APOA1s. These were of low abundance and were not analyzed here). Our current study extended this by utilizing calibrated ion mobility analysis and isotope dilution MS/MS with isotope-labeled APOA1 to quantify the absolute concentration of HDL particles and APOA1. This permitted, for the first time, the direct calculation of the number of APOA1 molecules per particle in the LpA-I^S^ and LpA-I^L^ fractions. Unexpectedly, LpA-I^L^ was consistently determined to contain only three APOA1s per particle vs. the four that were predicted (41). The LpA-I^S^ particles contained 3–4 APOA1s, generally in line with Segrest's predictions. In one experiment, we found the LpA-I^S^ number to be closer to 3, and in a second it was closer to 4 giving an average of 3.7, not a round number. The reason for variation in LpA-I^S^ is not clear. However, one explanation is that some LpA-I^S^ particles may contain a fourth APOA1 molecule that may be transiently interacting, i.e., not fully unfolded and engaging lipid with all its amphipathic helices. As it is not fully engaged in the integral trefoil scaffold of the particle, this fourth APOA1 molecule may act more like the low-abundance accessory proteins, which do not appear to impact the overall particle size. This is consistent with the observations of Lund-Katz *et al.* (57), who postulated a conformationally distinct population of transiently associated APOA1 molecules in small HDL particles from surface plasmon resonance data. Additionally, proteolysis experiments suggest that a limited fraction of APOA1 in LpA-I^S^ particles is more susceptible to exogenous proteases than APOA1 in LpA-I^L^ particles (not shown).

### APOA2 disrupts the formation of discrete sizes of HDL by APOA1

When APOA2 is present with APOA1 on HDL particles, as in total HDL isolated from plasma by ultracentrifugation or in LpA-I/A-II particles (supplemental Fig. S1), the paradigm of discrete particle size classes created by the APOA1 cage structure in LpA-I particles is disrupted. APOA2 may accomplish this by interfering with intermolecular salt-bridge interactions responsible for the rigidity of the APOA1 cage. This disruption could allow APOA1 helices to slide past each other to facilitate a much wider universe of stable particle diameters, accounting for the broad smear of LpA-I/A-II particles seen by native PAGGE analysis. This APOA2-induced alteration in the APOA1 scaffold is intriguing as it has implications not only for HDL particle diameter, but also for the activity of a host of HDL modifying factors. For example, lecithin: cholesterol acyl transferase (LCAT) appears to interact with a discontinuous epitope comprised of two APOA1 molecules in a double belt (58) and, presumably, in a trefoil. If APOA2 causes a disruption in APOA1 alignment in HDL, this could explain why APOA2 inhibits LCAT activity. We are currently exploring the impact of APOA2 on APOA1 structure and function.

### LpA-I particle composition and geometry

**Fig. 7:**
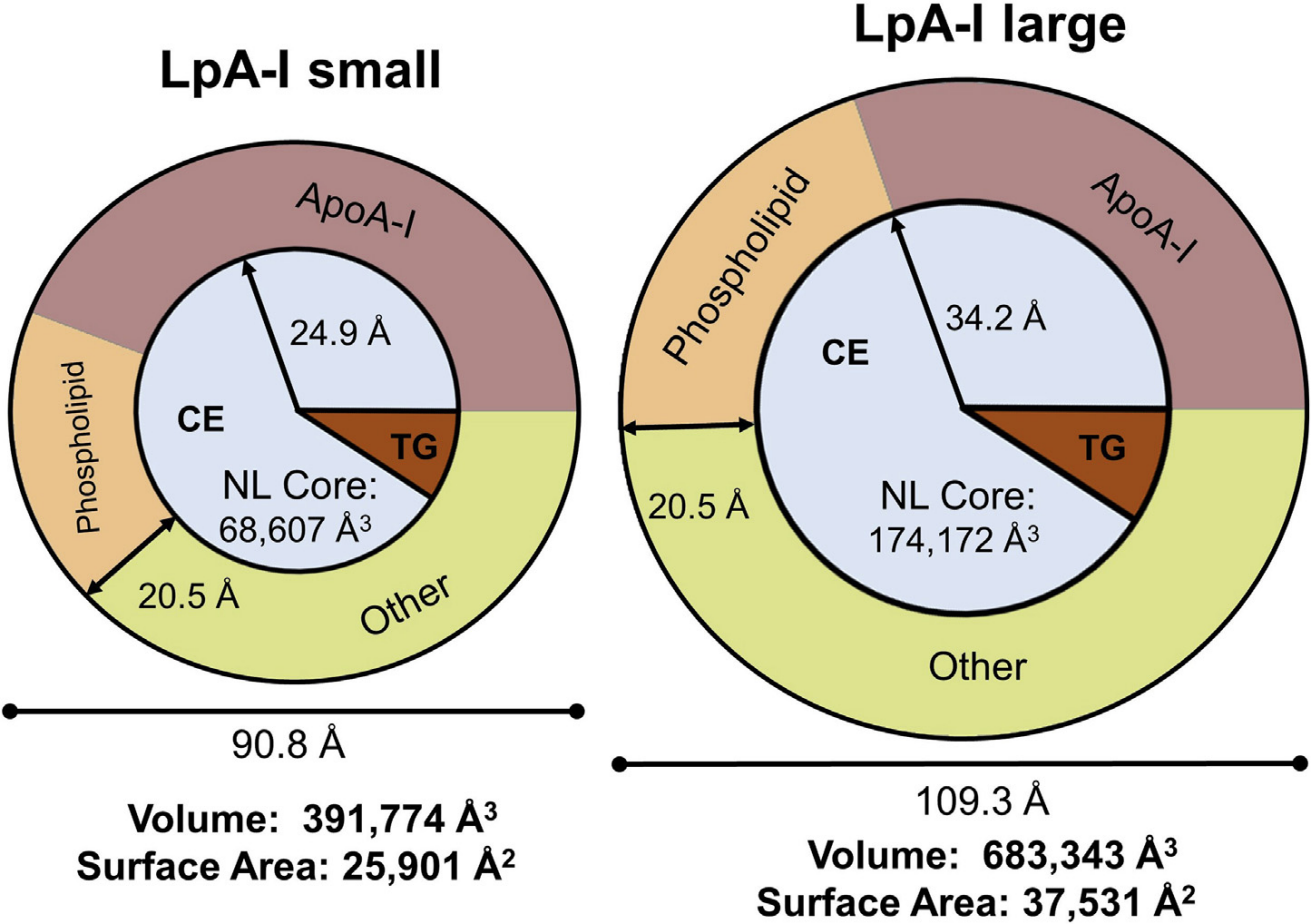
Volumetric and surface area analysis of LpA-I^S^ and LpA-I^L^. Schematic diagrams of the lipoprotein particles are shown, drawn approximately to scale. Values were calculated as described in Materials and Methods and supplemental data using the composition and dimensional results from triplicate preparations of particles from the 52-year-old donor. The coloring of the surface monolayer represents the calculated surface area occupied by various surface components. “Other” refers to space available for occupation by the minor HDL accessory proteins. The neutral lipid ester (NL) core consists of cholesteryl ester (CE) and triglyceride (TG) in light blue and orange, respectively.

We took pains to derive robust measurements of LpA-I particle diameters *via* EM and calibrated IMA. Applying the principles of the “oil drop” model of lipoproteins by Scanu and colleagues, we analyzed the geometry of LpA-I^S^ and LpA-I^L^ particles (Fig. 7) using the composition numbers from the three independent preparations from the 52-year-old donor. The molecular volumes of LpA-I^S^ and LpA-I^L^ were 392,000 Å^3^ and 683,000 Å^3^, giving a surface area of 26,000 Å^2^ and 38,000 Å^2^, respectively. Assuming a surface monolayer thickness of 20.5 Å (40), we estimated the volume of the neutral lipid ester core. For LpA-I^L^, the space available in the core matched closely with the space required for the number of CE and TG molecules that we measured per particle (assuming three molecules of APOA1 per particle). For LpA-I^S^, the calculated neutral lipid ester core volume was about 33% smaller than would be expected given CE and TG measurements, even if we assumed only three APOA1 molecules per particle. The reason for this is not clear, though it is possible that the core packing densities or surface layer thickness may be different in these particles.

Based on the number of APOA1 molecules per particle, we estimated the surface area occupied by the polar components of the lipoproteins (Fig. 7). For LpA-I^S^, we estimate that APOA1 and surface phospholipids account for about 62% of particle surface area. This assumes fully compressed APOA1 molecules (Materials and Methods). It is possible that more relaxed surface pressure conditions could allow APOA1 to cover more area. Nevertheless, this leaves a maximum of about 38% (or ∼9,950 Å^2^) of free particle surface area as potential binding sites for lipophilic accessory proteins listed in Fig. 5. LpA-I^L^, on the other hand, has 50% of its surface (or ∼18,700 Å^2^) free for accessory proteins. The fact that LpA-I^L^ has nearly double the free surface area likely accounts for our observed higher proteomic diversity in LpA-I^L^ vs. LpA-I^S^. Differences in surface curvature and surface pressure characteristics between the particles probably dictate the segregation of particular proteins between the subfractions noted in Fig. 6. Additionally, the attendant changes in APOA1 conformation may also favor or inhibit the docking of certain accessory proteins in each subfraction.

### Compositional differences between the subfractions

Our lipidomic analyses showed that the lipid complement carried by both subfractions is overall similar. With respect to the surface lipids, this is consistent with the notion that constant exchange in circulation keeps the lipid complements relatively homogeneous. However, we did find a few exceptions. There was a clear enrichment in sphingomyelin in the LpA-I^L^ population. Sphingomyelin is known to be enriched in lipid rafts on the cell surface and plays a well-known role in apoptosis (59). Interestingly, the LpA-I^L^ subfraction was also enriched in proteins involved in apoptosis possibly indicating functional relationship between the accessory proteins and accompanying lipids. The LpA-I^L^ enrichment of sphingomyelin also fits well with the tendency of these particles to contain more free cholesterol by weight as sphingomyelin is known to have a high affinity for free cholesterol (60).

In future studies, it will be important to determine the consequences of different particle sizes and compositions on the functions of LpA-I. We recently found that a large subfraction of HDL was strongly inversely associated with vascular stiffness in lean and obese adolescents (61). Intriguingly, these large HDLs exhibit an identical retention volume by SEC as the LpA-I^L^ particles studied here. As LpA-I/A-II HDL particles tend to be smaller in size as a whole, these LpA-I^L^ particles may be a strong candidate for the species we identified in adolescents. Current work is underway to confirm this as well as ascertain whether they represent biomarkers of a healthy vasculature or if they perhaps play an active beneficial role.

## Data availability

The mass spectrometry proteomics data have been deposited to the ProteomeXchange Consortium *via* the PRIDE (62) partner repository with the dataset identifier PCD026686.

## Supplemental Data

This article contains supplemental data (30).

## Conflict of interest

The authors declare that they have no conflicts of interest with the contents of this article.
