# Supplementary material for: Apolipoprotein A-I modulates HDL particle size in the absence of apolipoprotein A-II
Source: J Lipid Res. 2021 Jul 27;62:100099. doi: 10.1016/j.jlr.2021.100099 (PMC8385444; doi:10.1016/j.jlr.2021.100099)
Supplement: Supplemental Data [file mmc1.docx]

**Online Supplement for:**

**Apolipoprotein A-I modulates HDL particle size in the absence of apolipoprotein A-II**

John T. Melchior, Scott E. Street, Tomas Vaisar, Rachel Hart, Jay Jerome, Zsuzsanna Kuklenyik, Noemie Clouet-Foraison, Carissa Thornock, Shimpi Bedi, Amy S. Shah, Jere P. Segrest, Jay W. Heinecke and W. Sean Davidson

***** Supplemental Tables 1-3 are in a stand alone Excel document available at the same location as this supplement**

**Supplemental Table 4:** Gene ontology enrichment analysis of LpA-I^L^ and LpA-I^S^ subfractions

| **GO Function** | **LpA-I^L^**  **(%)** | **LpA-I^S^**  **(%)** | **P-Value** |
| --- | --- | --- | --- |
| **Acute inflammatory response** | 12.68  (3.20) | 15.33  (5.45) | 0.7831 |
| **Acute-phase response** | 7.99  (3.76) | 9.88  (4.15) | 0.7371 |
| **Complement activation** | 36.88  (8.84) | 35.18  (6.84) | 0.3855 |
| **Hemostasis** | 4.53  (2.09) | 5.45  (3.83) | 0.6548 |
| **Immune response** | 43.87  (10.02) | 47.14  (7.31) | 0.6918 |
| **Lipid metabolic process** | 63.22  (10.85) | 54.02  (9.50) | 0.1246 |
| **Lipid transport** | 40.94  (7.91) | 34.48  (8.44) | 0.1534 |
| **Platelet activation** | 2.54  (1.42) | 2.87  (2.36) | 0.5919 |
| **Proteolysis** | 1.79  (0.49) | 0.26  (0.14) | **0.0005** |
| **Reproduction** | 0.83  (0.82) | 1.17  (1.40) | 0.6576 |
| **Anti-oxidant activity** | 12.03  (3.95) | 13.12  (4.59) | 0.6344 |
| **Metal ion binding** | 20.81  (4.10) | 21.23  (7.82) | 0.5357 |
| **Peptidase inhibitor activity** | 3.26  (1.37) | 4.17  (0.99) | 0.8387 |
| **Apoptosis** | 23.57  (2.70) | 11.14  (5.97) | **0.0046** |

The abundance of proteins within each GO function was normalized to total protein abundance in the sample after removal of APOA1. Values represent the mean (± SD) of LpA-I^L^ and LpA-I^S^ for four independent isolations across the same donor. The P-value represents the statistical difference as determined by a paired student t-test.

**Supplemental Table 5:** Gene Ontology assignments of proteins identified in LpA-I^L^ and LpA-I^S^ subfractions.

| **Gene names** | **acute inflammatory response** | **acute-phase response** | **complement activation** | **hemostasis** | **immune response** | **lipid metabolic process** | **lipid transport** | **Platelet Activation** | **proteolysis** | **Reproduction** | **Anti-Oxidant Activity** | **metal ion binding** | **peptidase inhibitor activity** | **Apoptosis** |
| --- | --- | --- | --- | --- | --- | --- | --- | --- | --- | --- | --- | --- | --- | --- |
| **APMAP** |  |  |  |  |  |  |  |  |  |  |  |  |  |  |
| **ORM2** | X | X |  |  | X |  |  |  |  |  |  |  |  |  |
| **SERPINA1** | X | X |  | X | X |  |  |  |  |  |  |  | X |  |
| **APOA1** |  |  |  |  |  | X | X |  |  |  |  |  |  |  |
| **APOA2** | X |  |  |  |  | X | X |  |  |  |  |  |  |  |
| **APOA4** |  |  |  |  | X | X | X |  |  |  | X | X |  |  |
| **APOB** |  |  |  |  |  | X | X |  |  | X |  |  |  |  |
| **APOC1** |  |  |  |  |  | X | X |  |  |  |  |  |  |  |
| **APOC2** |  |  |  |  |  | X | X |  |  |  |  |  |  |  |
| **APOC3** |  |  |  |  |  | X | X |  |  |  |  |  |  |  |
| **APOC4** |  |  |  |  |  | X |  |  |  |  |  |  |  |  |
| **APOD** |  |  |  |  |  | X |  |  |  |  |  |  |  |  |
| **APOE** |  |  |  |  |  | X | X |  |  |  | X | X |  | X |
| **APOF** |  |  |  |  |  | X | X |  |  |  |  |  |  |  |
| **APOL1** |  |  |  |  | X | X | X |  |  |  |  |  |  |  |
| **APOM** |  |  |  |  |  | X | X |  |  |  | X |  |  |  |
| **C4BPA** |  |  | X |  | X |  |  |  |  |  |  |  |  |  |
| **CP** |  |  |  |  |  |  |  |  |  |  |  | X |  |  |
| **CLU** |  |  | X |  | X | X | X |  |  |  |  |  |  | X |
| **F5** |  |  |  | X |  |  |  |  |  |  |  | X |  |  |
| **C1QB** |  |  | X |  | X |  |  |  |  |  |  |  |  |  |
| **C1R** |  |  | X |  | X |  |  |  | X |  |  | X |  |  |
| **C1S** |  |  | X |  | X |  |  |  |  |  |  | X |  |  |
| **C3** |  |  | X |  | X | X |  |  |  |  |  |  | X |  |
| **C4B** |  |  | X |  | X |  |  |  |  |  |  |  | X |  |
| **CFH** |  |  | X |  | X |  |  |  |  |  |  |  |  |  |
| **FGA** |  |  |  | X | X |  |  | X | X |  |  | X |  | X |
| **FGB** |  |  |  | X | X |  |  | X | X |  |  |  |  | X |
| **FGG** |  |  |  | X |  |  |  | X | X |  |  | X |  | X |
| **FN1** | X | X |  |  |  |  |  |  |  |  |  |  |  |  |
| **FBLN1** |  |  |  | X |  |  |  |  |  | X |  | X |  |  |
| **HP** | X | X |  |  | X |  |  |  |  |  | X |  |  |  |
| **HPR** | X |  |  |  |  |  |  |  |  |  |  |  |  |  |
| **HBA1** |  |  |  |  |  |  |  |  |  |  |  | X |  |  |
| **IGHA1** |  |  | X |  | X |  |  |  |  |  |  |  |  |  |
| **IGHG2** |  |  | X |  | X |  |  |  |  |  |  |  |  |  |
| **IGHG3** |  |  | X |  | X |  |  |  |  |  |  |  |  |  |
| **IGHG4** |  |  | X |  | X |  |  |  |  |  |  |  |  |  |
| **HV307** |  |  | X |  | X |  |  |  |  |  |  |  |  |  |
| **IGKC** |  |  | X |  | X |  |  |  |  |  |  |  |  |  |
| **KV311** |  |  | X |  | X |  |  |  |  |  |  |  |  |  |
| **IGHM** |  |  | X |  | X |  |  |  |  |  |  |  |  |  |
| **ITIH1** |  |  |  |  |  |  |  |  |  |  |  | X | X |  |
| **ITIH2** |  |  |  |  |  |  |  |  |  |  |  |  | X |  |
| **KNG1** |  |  |  | X | X |  |  |  |  |  |  | X | X | X |
| **GPLD1** |  |  |  |  |  | X |  |  |  |  |  |  |  | X |
| **PLTP** |  |  |  |  |  | X | X |  |  |  |  |  |  |  |
| **KLKB1** | X |  |  | X |  |  |  |  | X |  |  |  |  |  |
| **PLXDC2** |  |  |  |  |  |  |  |  |  |  |  |  |  |  |
| **PCYOX1** |  |  |  |  |  |  |  |  | X |  |  |  |  |  |
| **AMBP** |  |  |  |  |  |  |  |  |  | X |  |  | X |  |
| **SFTPB** |  |  |  |  |  | X |  |  |  |  |  |  |  |  |
| **ALB** |  |  |  |  |  |  |  |  |  |  | X | X |  | X |
| **SAA1** | X | X |  | X | X |  |  | X |  |  |  |  |  |  |
| **SAA4** | X | X |  |  |  |  |  |  |  |  |  |  |  |  |
| **PON1** |  |  |  |  |  | X |  |  |  |  |  | X |  |  |
| **PROS1** |  |  |  | X |  |  |  |  |  |  |  | X | X |  |

**Supplemental Figure 1**

**
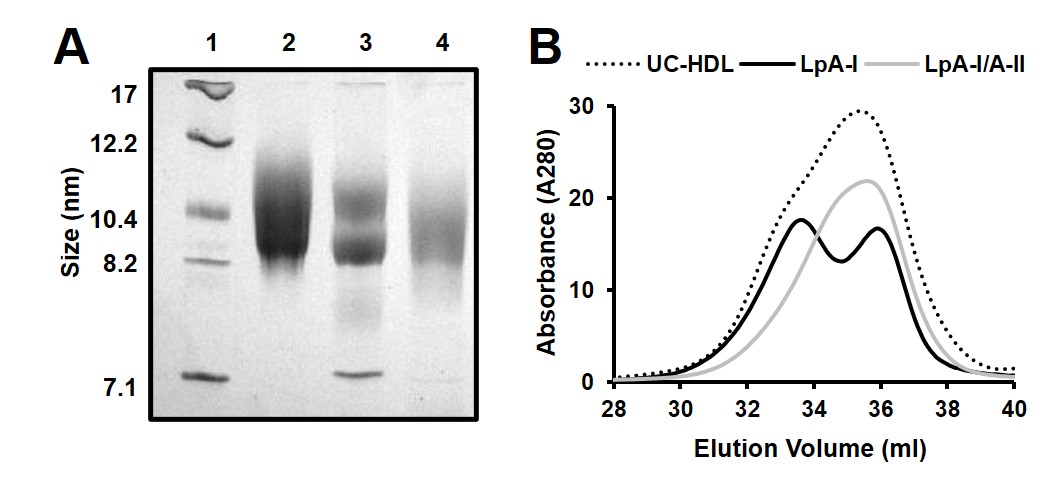
**

**Supplemental Figure 1:** **Size distribution HDL isolated by sequential ultracentrifugation (UC) and it’s LpA-I and LpA-I/A-II subfractions.** This panel was previously published (*Melchior et. al.^1^*) and is reproduced here for convenience**. Panel (A):** Equal mass of protein was loaded onto a native PAGGE and visualized by staining with Coomassie. Lane 1: Protein standards, lane 2: UC-HDL, lane 3: LpA-I from UC-HDL, lane 4: LpA-I/A-II from UC-HDL. **Panel (B):** UC-HDL and the LpA-I and LpA-I/A-II subfractions were separated using our traditional SEC setup containing three Superdex 200 columns in series in PBS at a flowrate of 0.3 ml/min.

**Supplemental Figure 2**

**
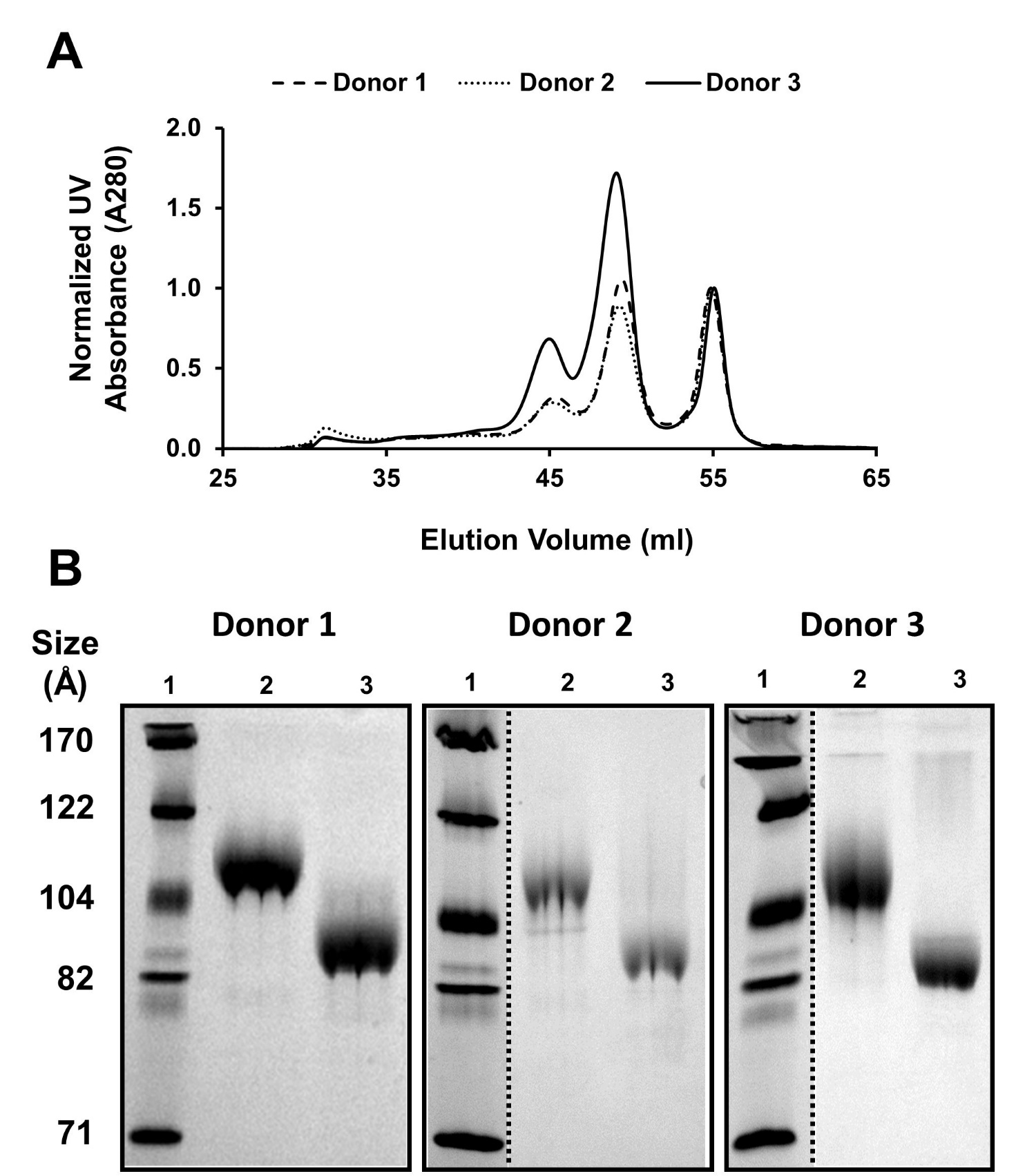
**

**Supplemental Figure 2:** **Size distribution LpA-I subfractions from three different anonymous donors of outdated plasma from the Hoxworth Blood Center.** **Panel (A):** The LpA-I subfractions was separated in PBS using four superdex columns in series at a flow rate of 0.15 ml/min. Peaks were normalized to the albumin peak across the individuals to control for different mass applied to the columns between individuals. **Panel (B):** Equal volumes of protein of isolated LpA-I^L^ and LpA-I^S^ sufractions were loaded and analyzed by native PAGGE. Lane 1: Protein standards, lane 2: LpA-I^L^, lane 3: LpA-I^S^. Protein was visualized by staining with Coomassie.

**Supplemental Figure 3**

**
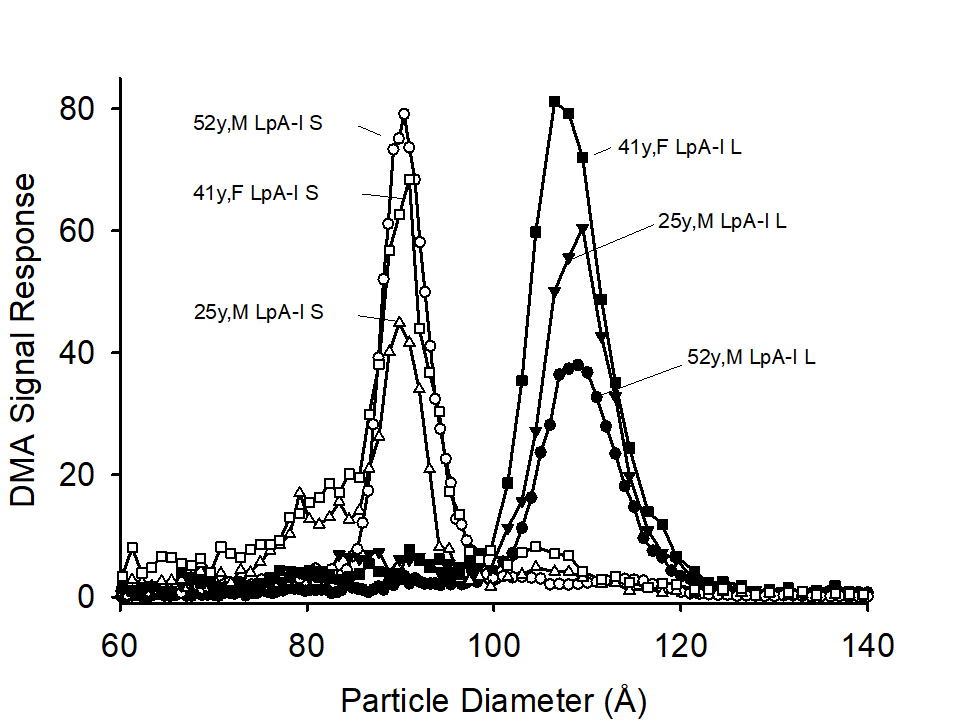
**

**Supplemental Figure 3: Quantification of particle size by from three fresh plasma donors by calibrated IMA.** LpA-I^L^ and LpA-I^S^ particles were analyzed calibrated ion mobility analysis (*Methods*) from three different subjects: 52 yo male (circles), 41 yo female (squares), and a 25 yo male (triangles). The LpA-I^L^ are filled and LpA-I^S^ are open. Traces are from one representative experiment on the 52 yo male (n=3 preparations).

**Supplemental Figure 4**

**
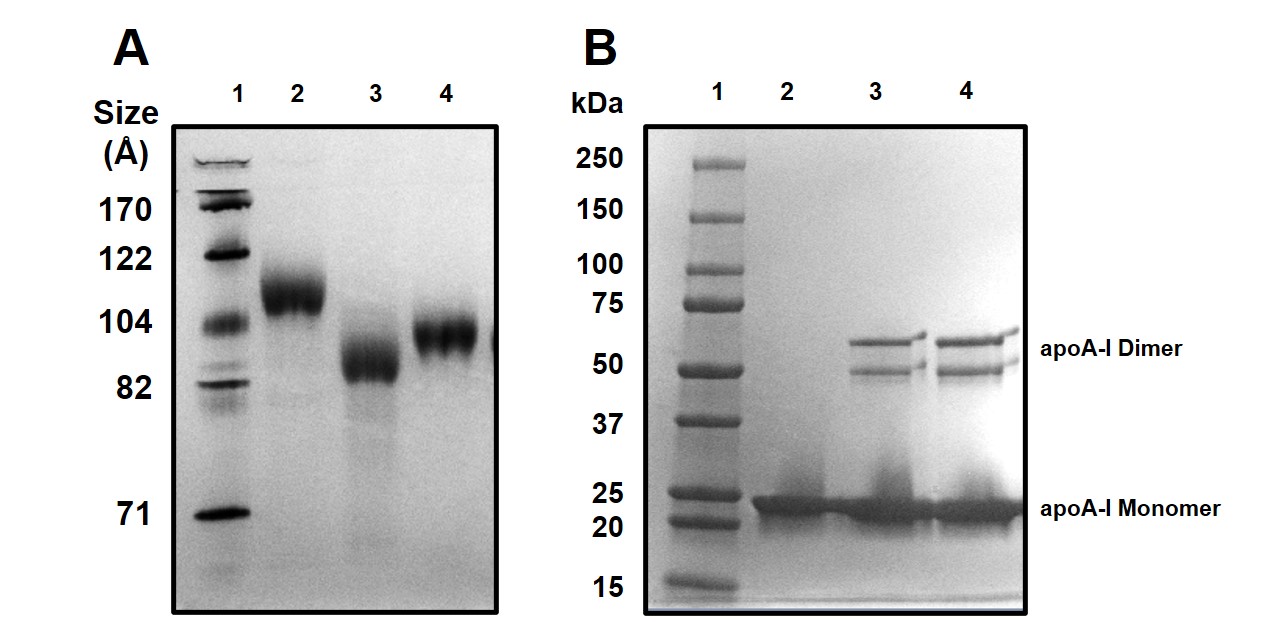
**

**Supplemental Figure 4: Analysis of rHDL control. Panel (A):** Native PAGGE analysis of plasma LpA-I subfractions and rHDL. Lane 1: Protein standards, Lane 2: LpA-I^L^, Lane 3: LpA-I^S^, Lane 4: rHDL generated with APOA1^.^ **Panel (B):** SDS-PAGE of rHDL cross-linked with BS^3^. Lane 1: Protein standards, Lane 2: rHDL with no cross-linker, Lane 3: rHDL cross-linked at 50:1 BS^3^:APOA1, lane 4: rHDL cross-linked at 100:1 BS^3^:APOA1. rHDL was cross-linked at a concentration of 0.5 mg/ml in PBS. Protein was visualized by staining with Coomassie.

1. Melchior JT, Street SE, Andraski AB, Furtado JD, Sacks FM, Shute RL, Greve EI, Swertfeger DK, Li H, Shah AS, Lu LJ and Davidson WS. Apolipoprotein A-II alters the proteome of human lipoproteins and enhances cholesterol efflux from ABCA1. *J Lipid Res*. 2017;58:1374-1385.
